# Enoxaparin-Induced Skin Necrosis

**Published:** 2016-09-21

**Authors:** James Coelho, David Izadi, Sameer Gujral

**Affiliations:** Department of Plastic Surgery, Royal Devon & Exeter Hospital, Exeter, Devon, England

**Keywords:** enoxaparin, skin necrosis, low-molecular-weight heparin, injection site, venous thromboembolic prophylaxis

**Figure:**
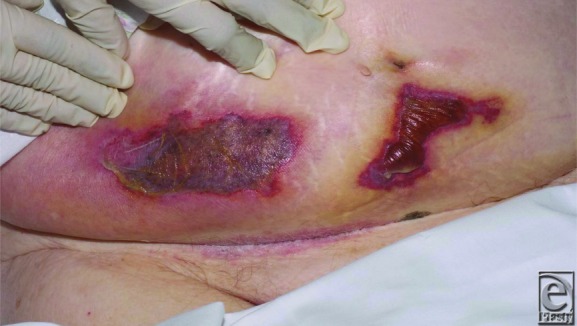


## DESCRIPTION

A previously well 96-year-old woman underwent a hemiarthroplasty of her right hip. She was treated postoperatively with subcutaneous injections of enoxaparin for venous thromboembolic (VTE) prophylaxis, as per hospital protocol. She developed 2 areas of skin necrosis at the location of the injection sites 4 days postoperatively.

## QUESTIONS

1. **What is the mechanism of action of enoxaparin?**
2. **What considerations need to be made when starting VTE prophylaxis?**
3. **What are the alternatives to low-molecular-weight heparin (LMWH) for surgical VTE prophylaxis?**
4. **Is this a common side effect of enoxaparin?**

## DISCUSSION

Enoxaparin is a commonly used LMWH in surgical VTE prophylaxis. It is administered as a subcutaneous injection. LMWH activates antithrombin III, which, in turn, inhibits coagulation factors Xa and to a lesser extent factor IIa. Factor Xa is essential in the conversion of prothrombin to thrombin; hence, inhibition of this coagulation factor prevents fibrin clot formation.^1^

VTE prophylaxis needs to be considered when patients are admitted into hospital, calculating the VTE risk versus the risk of bleeding for the individual patient. In the United Kingdom, surgical patients are considered to have a high VTE risk if they fulfill at least one of the following criteria^2^:

- Surgical procedure with a total anesthetic and surgical time of more than 90 minutes, or 60 minutes if the surgery involves the pelvis or lower limb.
- Acute surgical admission with inflammatory or intra-abdominal condition.
- Expected significant reduction in mobility.
- One or more of the risk factors shown in Box 1.

**Box 1. *Risk factors for VTE***

Active cancer or cancer treatment
Age >60 y
 Critical care admission
 Dehydration
 Known thrombophilias
 Obesity (body mass index >30 kg/m^2^)
 One or more significant medical comorbidities (eg, heart disease; metabolic, endocrine, or respiratory pathologies; acute infectious diseases; inflammatory conditions)
 Personal history or first-degree relative with a history of VTE
 Use of hormone replacement therapy
 Use of estrogen-containing contraceptive therapy
 Varicose veins with phlebitis

VTE indicates venous thromboembolic.

Pharmacological VTE prophylaxis is contraindicated if the patient fulfills 1 or more of the bleeding risks (Box 2):

**Box 2. *Risk factors for bleeding***

Active bleeding
Acquired bleeding disorders (such as acute liver failure)
 Concurrent use of anticoagulants known to increase the risk of bleeding (such as warfarin with international normalized ratio >2)
 Lumbar puncture/epidural/spinal anesthesia expected within the next 12 h
 Lumbar puncture/epidural/spinal anesthesia within the previous 4 h
 Acute stroke
 Thrombocytopenia (platelets <75 × 10^9^/L)
 Uncontrolled systolic hypertension (≥ 230/120 mm Hg)
 Untreated inherited bleeding disorders (such as hemophilia and von Willebrand disease)

VTE prophylaxis can be mechanical, pharmacological, or both. Mechanical prophylaxis includes:

- Antiembolic stockings (graduated compression stockings, with pressures of 14-18 mm Hg at the ankle and 14-15 mm Hg over the calf).
- Foot impulse devises.
- Intermittent pneumatic compression devices.

Alternatives to LMWH for VTE prophylaxis include:

- Fondaparinux sodium (another factor Xa inhibitor).
- Unfractionated heparin (used in patients with severe renal impairment).

Skin necrosis is a rare complication of LMWH, with just over 25 cases described in the literature.^3^ Three likely mechanisms causing the necrosis exist: immunologically mediated intravascular thrombosis resulting from heparin-induced immune aggregation of platelets (heparin-induced thrombocytopenia syndrome, HIT), formation of antigen-antibody complexes in cutaneous blood vessels (type III hypersensitivity syndrome), and, finally, the LMWH persisting in the subcutaneous tissue, due to poor circulation within the adipose tissue.^4^ The onset of skin necrosis typically occurs 5 to 11 days following the initiation of the LMWH, beginning as painful, erythematous, demarcating plaques. These progress rapidly into purpuric plaques and necrosis.^5^ Protein C and S deficiencies in patients greatly increase the risk of skin necrosis,^6^ and these should be measured if skin necrosis following enoxaparin injection is encountered.

Although enoxaparin-induced skin necrosis is rare, patients who have been affected by this rare drug reaction have gone on to develop severe and in some cases life-threatening complications. It is essential that clinicians, especially plastic surgeons, are able to recognize and address the skin necrosis early, caused by this commonly used medication. Plastic surgeons are likely to be involved in the initial assessment of these patients and possible subsequent debridement and reconstruction, where appropriate.
